# LitAutoScreener: Development and Validation of an Automated Literature Screening Tool in Evidence-Based Medicine Driven by Large Language Models

**DOI:** 10.34133/hds.0322

**Published:** 2025-09-02

**Authors:** Yiming Tao, Xuehu Li, Zuhar Yisha, Sihan Yang, Siyan Zhan, Feng Sun

**Affiliations:** ^1^ Key Laboratory of Epidemiology of Major Diseases, Ministry of Education/Department of Epidemiology and Biostatistics, School of Public Health, Peking University, Beijing, China.; ^2^ School of Cybersecurity, Hainan University, Hainan, China.; ^3^ School of Public Health, Peking University, Beijing, China.; ^4^ Research Center of Clinical Epidemiology, Peking University Third Hospital, Beijing, China.; ^5^ Center for Intelligent Public Health, Institute for Artificial Intelligence, Peking University, Beijing, China.; ^6^ Xinjiang Medical University Institute of Traditional Chinese Medicine, Urumqi 830017, China.; ^7^ School of Medicine, Shihezi University, Shihezi 832000, China.; ^8^ Department of Ophthalmology, Peking University Third Hospital, Beijing, China.

## Abstract

**Background:** The traditional manual literature screening approach is limited by its time-consuming nature and high labor costs. A pressing issue is how to leverage large language models to enhance the efficiency and quality of evidence-based evaluations of drug efficacy and safety. **Methods:** This study utilized a manually curated reference literature database—comprising vaccine, hypoglycemic agent, and antidepressant evaluation studies—previously developed by our team through conventional systematic review methods. This validated database served as the gold standard for the development and optimization of LitAutoScreener. Following the PICOS (Population, Intervention, Comparison, Outcomes, Study Design) principles, a chain-of-thought reasoning approach with few-shot learning prompts was implemented to develop the screening algorithm. We subsequently evaluated the performance of LitAutoScreener using 2 independent validation cohorts, assessing both classification accuracy and processing efficiency. **Results:** For respiratory syncytial virus vaccine safety validation title–abstract screening, our tools based on GPT (GPT-4o), Kimi (moonshot-v1-128k), and DeepSeek (deepseek-chat 2.5) demonstrated high accuracy in inclusion/exclusion decisions (99.38%, 98.94%, and 98.85%, respectively). Recall rates were 100.00%, 99.13%, and 98.26%, with statistically significant performance differences (*χ*^2^ = 5.99, *P* = 0.048), where GPT outperformed the other models. Exclusion reason concordance rates were 98.85%, 94.79%, and 96.47% (*χ*^2^ = 30.22, *P* < 0.001). In full-text screening, all models maintained perfect recall (100.00%), with accuracies of 100.00% (GPT), 100.00% (Kimi), and 99.45% (DeepSeek). Processing times averaged 1 to 5 s per article for title–abstract screening and 60 s for full-text processing (including PDF preprocessing). **Conclusions:** LitAutoScreener offers a new approach for efficient literature screening in drug intervention studies, achieving high accuracy and significantly improving screening efficiency.

## Introduction

In modern medicine, evidence-based medicine plays a pivotal role. It integrates the best available evidence with clinical expertise and patient values to guide medical decision-making [1,2]. Systematic reviews, a cornerstone of evidence-based medicine, systematically collect, evaluate, and synthesize relevant research data, providing an objective foundation for medical decisions and finding extensive applications in public health [3–5].

Drug intervention studies are crucial for enhancing patient health and refining medical practices, given the direct impact of drug efficacy and safety on patient outcomes [6]. Pre-market randomized controlled trials are invaluable, providing initial assessments of drug efficacy and safety under controlled conditions, which are essential for drug approval and integral to the drug development process [7,8]. Post-market real-world studies are equally important, enabling research in natural healthcare settings across diverse patient populations, identifying potential drug-related issues, and supporting rational drug use and optimization [9,10].

Conducting systematic reviews of drug intervention studies requires researchers to sift through extensive literature [11]. Traditional screening methods, which are reliant on manual review, are not only inefficient but also susceptible to subjective biases, resulting in inconsistent and nonreproducible outcomes [12,13]. Although automated screening methods, which leverage machine learning and deep learning, have enhanced efficiency and objectivity, current tools exhibit limitations. These tools often merely decide on literature inclusion or exclusion without justifying exclusion decisions, fail to adhere to Preferred Reporting Items for Systematic Reviews and Meta-Analyses (PRISMA) reporting standards, and are typically confined to title and abstract screening, lacking full-text screening capabilities [14–16].

Large language models (LLMs), which exhibit advanced linguistic knowledge and semantic comprehension capabilities due to their exceptional performance in natural language processing [17], have been widely adopted across various disciplines by researchers, practitioners, and industry professionals [18–20]. Prompt learning, an innovative approach, enhances the performance of pretrained models on specific tasks. It is characterized by its flexibility, efficiency, and interpretability, and has applications in healthcare [21].

This study integrates LLMs with prompt learning strategies to develop a novel automated literature screening tool, LitAutoScreener. The tool aims to streamline the title–abstract screening process of drug intervention study literature, enable full-text screening, enhance screening accuracy and efficiency, expedite systematic review processes, provide more reliable literature support for drug intervention studies, and thereby furnish a robust scientific basis for clinical decision-making.

## Methods

### Data sources

The research data utilized for the development of the tool were primarily derived from literature databases on vaccines, hypoglycemic drugs, and antidepressants that had been previously established by our team. Manual screening and information extraction ensured the accuracy and reliability of the data, thereby establishing the gold standard for the development and optimization of the tool. Two trained researchers independently conducted literature screening and extraction, strictly adhering to predefined protocols. Any discrepancies or controversial cases were resolved through discussion or consultation with a third-party expert. Prior to training, all manually screened and extracted data were rechecked to verify their accuracy.

A substantial proportion of the literature was excluded due to failure to meet the inclusion criteria. In the gold standard database, the manually determined inclusion-to-exclusion ratio was 1:9. Data stratification was conducted accordingly to balance computational resources, training time, and performance. Drawing on prior studies, we finalized a training set comprising 400 articles (40 included and 360 excluded). This 1:9 stratification ensured that the training set contained an adequate amount of relevant literature for the model to learn key features, while also exposing it to excluded literature, thereby enhancing its accuracy in preliminary relevance assessments. Certain literature erroneously passed the preliminary screening. Based on the actual ratio of the gold standard, the data stratification was adjusted to 3:1 (included:excluded). In order to balance sample adequacy and computational efficiency, the training set consisted of 80 articles (60 included and 20 excluded). This phase entailed an in-depth analysis of the preliminarily eligible literature to confirm its adherence to the inclusion criteria.

### Data preprocessing

In the title–abstract preprocessing stage, key information such as publication time, authors, and titles is retrieved from the literature database. Text-cleaning techniques are employed to eliminate irrelevant characters. Duplicate documents are identified and removed by comparing key information. The processed documents are then organized into XML format, providing standardized data for model training and analysis.

In the PDF full-text preprocessing process, the core task is to accurately extract key components such as the title, abstract, and methodology sections from the PDF files of English papers. First, a common subtitle vocabulary library is established for identification and segmentation. Subtitles are identified by leveraging text features, and adjacent texts are merged. Table and picture elements are recognized, and the information of each part is mapped, sorted, classified, and stored to generate a structured article. For full-texts in picture format, optical character recognition (OCR) technology is utilized to convert them into an identifiable text format for subsequent research and analysis.

### Development of LitAutoScreener

**Fig. 1.**
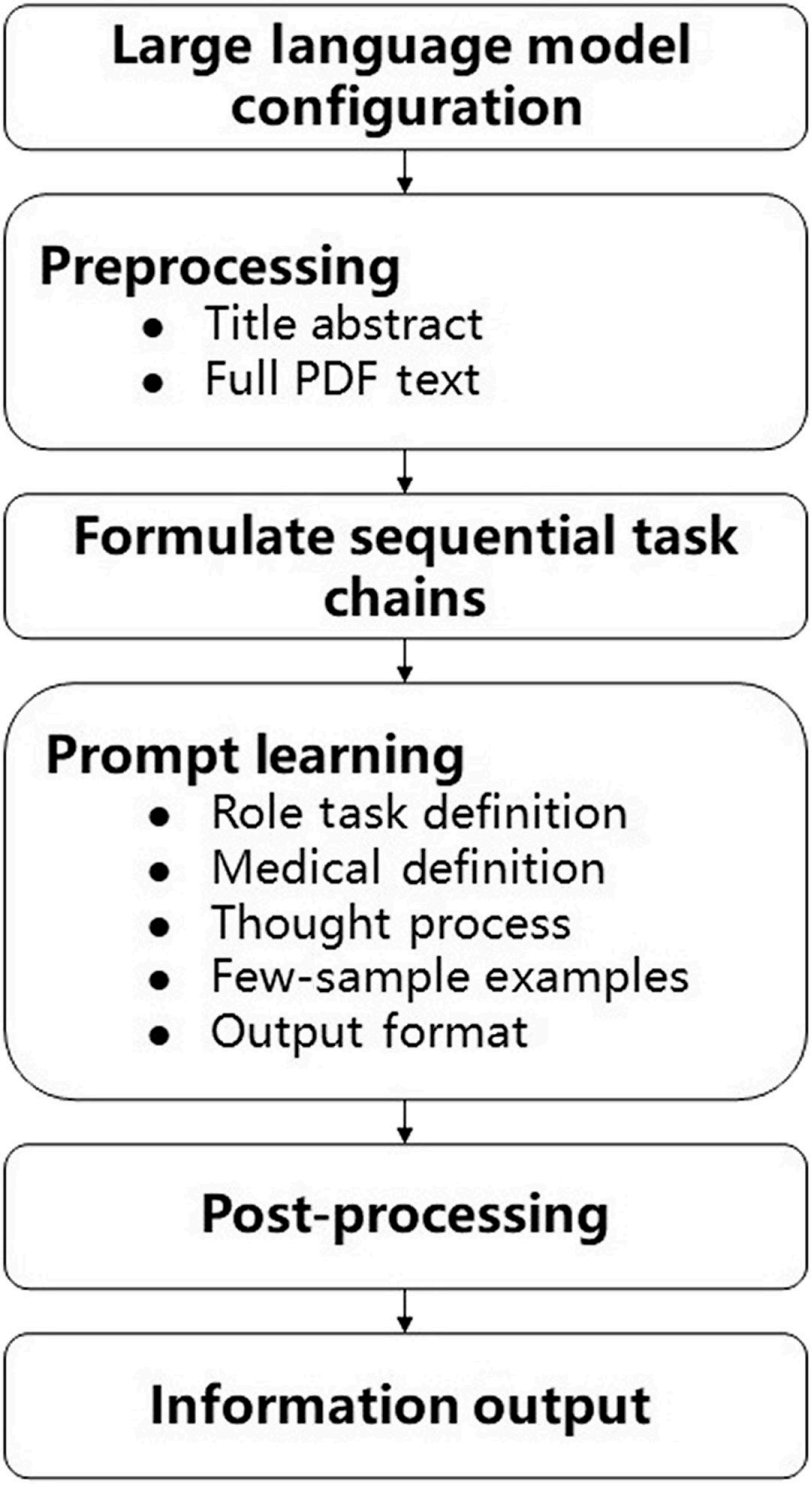
Tool development process.

The tool development process is depicted in Fig. 1. The screening tool implements chained tasks within the PICOS (Population, Intervention, Comparison, Outcomes, Study Design) framework, systematically decomposing the literature screening process into subtasks that correspond to specific exclusion criteria. The PICOS framework, which is widely adopted in medical research, identifies 5 key components of research questions: Population, Intervention, Comparison, Outcome, and Study Design. For each PICOS dimension, we established detailed exclusion criteria that are aligned with our specific research objectives. For instance, when evaluating the efficacy of a new antihypertensive drug in patients with primary hypertension, we defined the following: for population—“study participants without primary hypertension”; for intervention—“treatment not using the specified antihypertensive agent”.

As detailed in Table 1, we employed few-shot prompt learning combined with chain-of-thought reasoning to replicate human screening logic based on PICOS-derived criteria. Our methodology proceeded through 3 phases: First, we developed a preliminary prompt framework using limited training data and incorporating representative examples to establish task understanding. Second, we progressively expanded to the complete training set while iteratively refining prompt components to enhance generalizability. Finally, we addressed persistent challenges by directly incorporating misclassified cases as few-shot examples, thereby enabling targeted performance optimization.

**Table 1.** Examples of automated screening prompt words

Prompt construction	Example content
Role task definition	As an expert in epidemiology and evidence-based medicine, familiar with drug intervention studies, you should determine the type of literature research, such as “clinical research”, “animal experiment”, or “basic experiment and research”, based on the title and abstract information of a piece of literature.
Medical definition	#Medical definition “Clinical research” generally refers to research conducted on human subjects to assess vaccine safety and efficacy and to determine the optimal dosage and vaccination schedule…
Thought process	#Step-by-step thinking Check if the research subjects are mentioned. For instance, if it describes human subjects and the trials, observations, or interventions on them, along with their effectiveness and safety results, it is classified as “clinical research”… Check if specific research methods and designs, such as randomized controlled trials, cohort studies, or case–control studies, are mentioned. If so, it is classified as “clinical research”… Check if the research purpose, such as evaluating vaccine safety, efficacy, and dose–response relationship in humans, is mentioned. If yes, it is classified as “clinical research”…
Few-sample examples	#Representative examples or examples of screening errors in the training and test sets If the research involves simulation experiments on cells, molecules, or under other laboratory conditions, the literature research type is classified as “basic experiment and research”… If the research is an experiment on an animal model prior to human clinical trials, the literature research type is classified as “animal experiment”…
Output format	#Standard output format The response must be in JSON format. The return format is as follows: “Type of literature research”: str

During the actual screening process, detailed exclusion criteria are formulated for each dimension in accordance with research requirements and inclusion criteria. For instance, if the inclusion criterion is the efficacy of a specific new antihypertensive drug on patients with essential hypertension, for the research-subject dimension, the exclusion criterion would be “Research subjects are not patients with essential hypertension”; for the intervention-measure dimension, it would be “Intervention measure is not the specific new antihypertensive drug”. In this manner, multidimensional and elaborate exclusion criteria are constructed to comprehensively evaluate the literature.

As detailed in Table 1, we employed few-shot prompt learning combined with chain-of-thought reasoning to replicate human screening logic based on PICOS-derived criteria [22–24]. Our methodology proceeded through 3 phases: First, we developed a preliminary prompt framework using limited training data and incorporating representative examples to establish task understanding. Second, we progressively expanded to the complete training set while iteratively refining prompt components to enhance generalizability. Finally, we addressed persistent challenges by directly incorporating misclassified cases as few-shot examples, thereby enabling targeted performance optimization.

### Verification of data sources

We evaluated the tool’s performance using 2 distinct clinical scenarios: (a) the safety assessment of RSV vaccines and (b) randomized controlled trials investigating antibody–drug conjugates (ADCs) in lung cancer patients (Supplementary Materials). These validation scenarios collectively encompassed all PICOS components. Notably, although the training set included vaccine studies, it contained no RSV vaccine research, thereby ensuring complete independence between the training and validation datasets.

For RSV Vaccine Safety Studies, the system conducted comprehensive searches in 5 English databases—Ovid, Scopus, Clinical trials, Web of Science, and Cochrane Library—as well as 4 Chinese databases: Wanfang Database, China National Knowledge Infrastructure (CNKI), SinoMed, and VIP. The search utilized “RSV+vaccine” and its synonyms as keywords, with the retrieval deadline set for 26 September 2024.

The inclusion criteria were as follows: (a) Research subjects: individuals who received the RSV vaccine. (b) Intervention measure: administration of the RSV vaccine. (c) Outcome indicators: safety-related outcomes, such as local adverse events at the injection site, systemic adverse events, and serious adverse events. (d) Research types: experimental and observational studies.

**Fig. 2.**
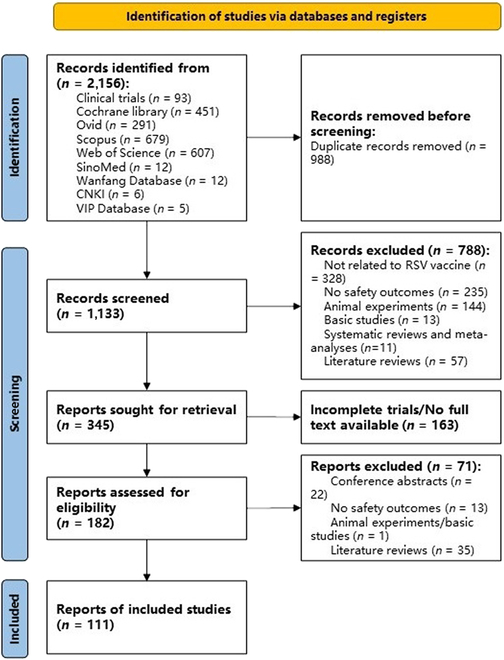
RSV vaccine literature inclusion and exclusion flow diagram by human reviewers.

In this search, researchers retrieved a total of 2,156 documents. After removing duplicates, 1,133 documents underwent title–abstract screening. During the screening process, 182 documents advanced to the full-text screening stage, among which 71 were excluded during this phase (Fig. 2).

### Evaluation methods

The results of manual screening in traditional systematic reviews serve as the gold standard for evaluating the performance of the tool. To comprehensively assess the overall performance of automated literature screening and information extraction tools, an in-depth analysis of the tools’ accuracy and efficiency will be conducted from both quantitative and qualitative perspectives.

#### Quantitative evaluation

During literature screening, to precisely quantify the tool’s performance in title–abstract and full-text screening, we selected classic evaluation indicators commonly used in deep learning, namely Accuracy, Precision, and Recall. These metrics comprehensively measure the accuracy and completeness of the tool’s screening results. Additionally, we used the consistency rate of literature exclusion reasons to evaluate the consistency between the model’s reasons for excluding literature and those from manual screening.

$$\text{Accuracy}=\frac{\mathrm{TP}+\text{TN}}{\text{TP}+\text{TN}+\text{FP}+\text{FN}}$$ (1)

$$\text{Precision}=\frac{\text{TP}}{\text{TP}+\text{FP}}$$ (2)

$$\text{Recall}=\frac{\text{TP}}{\text{TP}+\text{FN}}$$ (3)

TP represents the number of actual positive samples predicted as positive by the tool (true positives); TN represents the number of actual negative samples predicted as negative (true negatives); FP represents the number of actual negative samples predicted as positive (false positives); and FN represents the number of actual positive samples predicted as negative (false negatives).

To compare the performance of tools developed by different LLMs, we evaluated the GPT model (GPT-4o) [25], Kimi model (moonshot-v1-128k) [26], and DeepSeek model (deepseek-chat 2.5) [27] on the RSV vaccine safety validation set under the low-randomness condition of temperature = 0. We compared and analyzed the performance of each tool on these indicators to identify their advantages and disadvantages, providing a basis for tool selection and facilitating the optimization of the literature screening process. Categorical data were presented as frequencies (constituent ratios), and the chi-square test was applied to determine if there were significant differences in the performance of different model indicators (α = 0.05). The statistical analyses were conducted using R 4.1.1.

#### Qualitative evaluation

We conducted a qualitative evaluation by systematically analyzing the output results of different tools, focusing on texts with incorrect inclusion–exclusion classification and mislabeled exclusion reasons. Through in-depth analysis of these errors, we aimed to uncover the tool’s strengths and weaknesses in screening drug-safety literature, as well as the challenges it faces in handling complex medical contexts and technical terms. These challenges include misinterpretations of technical terms, misunderstandings of contextual semantics, and improper handling of special sentence structures. Based on this analysis, we identified areas for tool improvement and provided targeted suggestions for model iteration and enhancement.

#### Time efficiency evaluation

We measured the time required for automated tools to perform both title–abstract screening and full-text screening. These measurements were subsequently compared with the estimated completion times for traditional systematic reviews, thereby quantitatively demonstrating the time savings achieved through automation.

## Results

### Inclusion–exclusion accuracy in title–abstract screening

We conducted systematic testing of automated literature screening tools developed using 3 distinct LLMs. As shown in Table 2, the evaluated models—GPT, Kimi, and DeepSeek—all exhibited high performance in title–abstract literature screening, with accuracy rates exceeding 98%. Specifically, GPT, Kimi, and DeepSeek produced incorrect inclusion or exclusion results for 7, 12, and 13 studies. Statistical analysis revealed significant differences in recall rates between the models (*χ*^2^ = 5.99, *P* = 0.048). The GPT model exhibited optimal performance with a recall rate of 100%, significantly outperforming the other models (Kimi: 99.13%; DeepSeek: 98.26%).

**Table 2.** Inclusion–exclusion accuracy in title–abstract screening

Model	Accuracy	Recall	Precision
GPT (GPT-4o)	99.38%	100.00%	98.01%
Kimi (moonshot-v1-128k)	98.94%	99.13%	96.88%
DeepSeek (deepseek-chat 2.5)	98.85%	98.26%	97.98%

### Consistency rate of exclusion reasons in title–abstract screening

Significant differences exist in the consistency rates of exclusion reasons in title–abstract screening among tools developed from different models (*χ*^2^ = 30.22, *P* < 0.001). Specifically, the GPT model achieved a consistency rate of 98.85%, the Kimi model achieved a consistency rate of 94.79%, and the DeepSeek model achieved a consistency rate of 96.47%. Even with the use of the chained-task approach, which may lead to an underestimation of the rate, the GPT model still demonstrates a high level of consistency with manual-screening results.

### Qualitative analysis of title–abstract screening results

Given the relatively low inclusion–exclusion recall rate of the DeepSeek model (deepseek-chat 2.5), we undertook a qualitative analysis of the potential causes (Table 3). In handling literature related to randomized controlled trial (RCT) studies, the DeepSeek model erroneously excluded some documents, presumably due to its inadequate comprehension of the research-design type. This likely stems from the model’s inaccurate grasp of RCT-study characteristics during its learning phase, failing to fully comprehend the essence and key elements of RCTs.

**Table 3.** Qualitative analysis of inclusion–exclusion errors in title–abstract screening

Error reason	Model	Manual annotation screening result	Example	Explanation
Poor understanding of research design type	DeepSeek	Inclusion	A randomized, double-blind, placebo-controlled study to evaluate the efficacy of a single immunization of AD26.RSV.Pref against RSV infection in a viral challenge model in healthy adults.	Because of the limited length of the RCT abstract, specific safety results may not be clearly mentioned. However, safety outcomes are generally reported in the full text. To avoid missing important literature, RCT studies of RSV vaccines will not be excluded during the screening stage merely because safety outcomes are not mentioned.

Moreover, as an abbreviated summary of the literature content, the abstract typically does not encompass all research details. Thus, when processing abstract information, the model should possess inferential capabilities to make reasonable speculations about the overall literature based on the available data. The DeepSeek model, however, may be deficient in this regard. It may overly focus on the explicitly stated content in the abstract, without fully considering the general characteristics and patterns of RCT studies, resulting in misjudgments of the literature.

There are errors in the annotation of literature exclusion reasons, specifically manifested as problems such as failure to locate the intervention measures, poor understanding of the research outcomes, and insufficient understanding of the original research (Table 4). This is primarily because the model has an inaccurate understanding of relevant concepts during the learning process, lacks an effective identification and judgment mechanism, overly relies on vocabulary matching, ignores semantic analysis, and cannot accurately identify when facing the concise and condensed text in the abstract as well as new and uncommon descriptions.

**Table 4.** Qualitative analysis of errors in annotating literature exclusion reasons

Error reason	Model	Manual annotation screening result	Example	Explanation
Failure to identify intervention measures	Kimi	Unrelated to RSV vaccine	The molecular epidemiology and characterization of HRSV strains detected at a Spanish tertiary hospital during the 2013–2014 season is reported.	This article focuses on the surveillance and analysis of RSV infection cases and does not mention the RSV vaccine.
Failure to identify intervention measures	Kimi, DeepSeek	Unrelated to RSV vaccine	An RSV monoclonal antibody (palivizumab) is currently available for passive immunoprophylaxis in high-risk infants.	This article describes an RSV-specific immunoglobulin, not an RSV vaccine.
Poor understanding of research outcomes	DeepSeek	Not a safety study of RSV vaccine	We investigated whether chitosan administered 1 or 3 days postinfection could protect animals against RSV infection and whether it could alter immune responses or immunopathology induced by inactivated RSV vaccine when administered twice before RSV infection.	Only the protective effect of the vaccine against the disease is mentioned in the abstract, which pertains to the effectiveness outcome rather than the safety outcome.
Poor understanding of the original research	GPT, DeepSeek	Literature review on the safety of RSV vaccine	Prophylaxis and treatment of RSV in infants using human immunoglobulin containing high titers of RSV-specific neutralizing antibody (RSV-Ig) has shown limited success in different infant populations.	It is difficult to determine whether the research results represent the author’s own findings or a summary of others’ research.

### Accuracy of full-text screening

Table 5 presents the evaluation metrics for full-text screening based on predefined inclusion/exclusion criteria. All 3 evaluated models—GPT, Kimi, and DeepSeek—achieved consistently high performance across all metrics.

**Table 5.** Inclusion–exclusion accuracy in full-text screening

Model	Accuracy	Recall	Precision
GPT	100.00%	100.00%	100.00%
Kimi	100.00%	100.00%	100.00%
DeepSeek	99.45%	100.00%	99.11%

### Time efficiency evaluation

The automated literature screening tool developed in this study exhibits significantly higher efficiency compared to manual processing. Although the tool’s performance is influenced by API configuration parameters, including timeout settings and maximum concurrent requests (owing to its cloud-based rather than local implementation), accurate quantitative benchmarking remains challenging. However, our empirical results demonstrate substantial efficiency gains. The tool is capable of screening the titles and abstracts of documents within 1 to 5 s and completing the full-text processing, including PDF preprocessing, in approximately 60 s per article. This represents a significant improvement over single-person manual screening, which typically requires 30 to 60 s for each title–abstract screening and 5 min for each full-text screening.

## Discussion

### Performance of LitAutoScreener in the literature screening task

We evaluated the literature screening performance of LitAutoScreener in 2 validation scenarios: RSV vaccine safety and RCTs of ADCs for lung cancer patients (Supplementary Materials). The results showed that at the title–abstract screening stage, the tool maintained an accuracy rate of over 98%, rapidly and accurately identifying literature irrelevant to the research topic. The accuracy exceeds the validation findings of Tran et al. [28], who used the general prompt framework within the GPT-3.5 Turbo model. Additionally, it is comparable to the validation results of Oami et al. [29], who employed the debugged prompt in the context of the GPT-4 Turbo model.

At the full-text screening stage, all 3 models maintained high accuracy and recall rates. This indicates that when conducting in-depth analysis of literature content, the tool can precisely grasp the core points of the literature and make accurate judgments based on the specific requirements of the research. The high accuracy rate ensures that the included literature is highly consistent with the research topic, effectively reducing interference from irrelevant literature and thereby laying a solid foundation for the subsequent step of literature information extraction. The diminished precision observed in the Kimi model during ADC drug validation scenarios primarily results from the erroneous inclusion of conference abstracts that deviate from standard 2-column formatting conventions (Table S3). This performance limitation underscores the necessity for additional algorithmic refinements to enhance the tool’s robustness when processing heterogeneous literature formats, particularly nonstandard document layouts that challenge current text parsing capabilities.

### Consistency analysis of exclusion rationales

The consistency rate of exclusion rationales during title–abstract literature screening serves as a pivotal metric for evaluating screening tool performance, directly reflecting the concordance between automated and manual exclusion criteria [30]. This metric provides essential evidence for both the interpretability of tool-generated results and the reproducibility of systematic reviews. High accuracy in inclusion/exclusion decisions demonstrates the tool’s capability to precisely identify and eliminate literature irrelevant to research objectives, while strong consistency in exclusion rationales indicates that the tool employs reasoning patterns analogous to those used in human screening. These characteristics significantly enhance the credibility of screening outcomes, offering researchers clear and reliable references to thoroughly comprehend the screening process and accurately assess the validity of results.

In practical screening scenarios, the situation is more complex, as individual articles may warrant exclusion for multiple reasons corresponding to various exclusion rationales. To address this complexity, our study implemented a chained evaluation pathway assessing 4 sequential domains: interventions/comparators, study populations, outcomes, and study designs, in accordance with predefined decision rules. While this structured approach enables systematic literature evaluation, its binary classification may conservatively estimate true consistency rates when articles present multiple exclusion grounds.

Qualitative analysis of model-generated rationales revealed 3 predominant error patterns (Table 4): failure to identify interventions, inadequate comprehension of study outcomes, and insufficient understanding of original research designs. These limitations primarily stem from imperfect conceptual learning during model training, ineffective identification mechanisms, overreliance on lexical matching at the expense of semantic analysis, and the consequent difficulties in accurately interpreting concise abstract texts containing novel or uncommon terminology.

### Advantages over existing tools

LitAutoScreener exhibits substantial improvements over existing tools for literature screening and information extraction. In the screening phase, current automated tools, such as Abstrackr [14], employ machine learning techniques to achieve partial automation but remain semiautomatic in practice, requiring extensive manual preprocessing before screening [31]. In contrast, our tool enables fully automated screening, from initial triage to full-text assessment, efficiently and accurately processing large volumes of literature without manual review. This not only reduces labor and time costs but also enhances the consistency and reliability of screening outcomes, thereby optimizing overall research efficiency.

Furthermore, existing tools often lack transparency in decision-making, offering only binary inclusion/exclusion judgments without documenting the rationale or reasoning. Such opacity impedes retrospective validation and compromises the evaluation of screening validity, thereby undermining the credibility of research findings. Our tool addresses this by generating detailed, traceable exclusion justifications for each excluded record, thereby ensuring researchers can scrutinize the basis for exclusion. This adherence to PRISMA guidelines for transparent and standardized screening bolsters the credibility and reproducibility of results, thereby establishing a robust foundation for downstream research [30].

In typical screening workflows, 2 critical stages exist: preliminary title–abstract screening and subsequent full-text evaluation. While current tools handle the former, they fail to automate the latter—a more decisive step [14,15]. LitAutoScreener bridges this gap with its advanced full-text screening capability, ensuring comprehensive coverage and delivering a more accurate literature corpus for subsequent extraction and analysis.

### Limitations

This study has several limitations. First, the application scenario of the developed screening tool is relatively narrow, applicable only to drug intervention studies. Its potential in other evidence-based medicine topic scenarios remains underexplored. To fully realize the tool’s potential, future research should focus on expanding its application scope to diverse evidence-based medicine scenarios, such as pharmacoeconomic research and lifestyle intervention research, thereby continuously pushing the boundaries of its application. Second, this study only validated 3 LLMs: GPT, Kimi, and DeepSeek. The performance of other models in the literature-screening task remains unknown. Future research could expand the range of model selection, evaluate, and compare more LLMs to identify a more suitable one for the literature-screening task. Finally, the integration with the entire evidence-based medicine process is insufficient. For other aspects of evidence-based medicine, such as formulating research questions, research design, information extraction, and analyzing and interpreting research results, the advantages of LLMs have not been fully exploited. Future research should target these aspects, develop corresponding tools and applications, and achieve seamless integration and collaborative operation of LLMs throughout the evidence-based medicine process, thereby further improving the efficiency and quality of evidence-based medicine research.

## Conclusion

An automated literature screening tool, LitAutoScreener, specifically designed for drug intervention studies, was developed using LLMs. This tool provides detailed reasons for excluding literature during the preliminary screening stage and performs refined screening based on the full-text PDF versions of documents. In the validation scenario, LitAutoScreener demonstrated high accuracy and efficiency, thereby significantly enhancing the conduct of drug intervention studies.

## Data Availability

The data that support the findings of this study are available from the corresponding author upon reasonable request.
