# Supplementary material for: LitAutoScreener: Development and Validation of an Automated Literature Screening Tool in Evidence-Based Medicine Driven by Large Language Models
Source: Health Data Sci. 2025 Sep 2;5:0322. doi: 10.34133/hds.0322 (PMC12404845; doi:10.34133/hds.0322)
Supplement: Supplementary 1 — Fig. S1 Tables S1 to S3 [file hds.0322.f1.zip › sm.docx]

Supplementary Materials


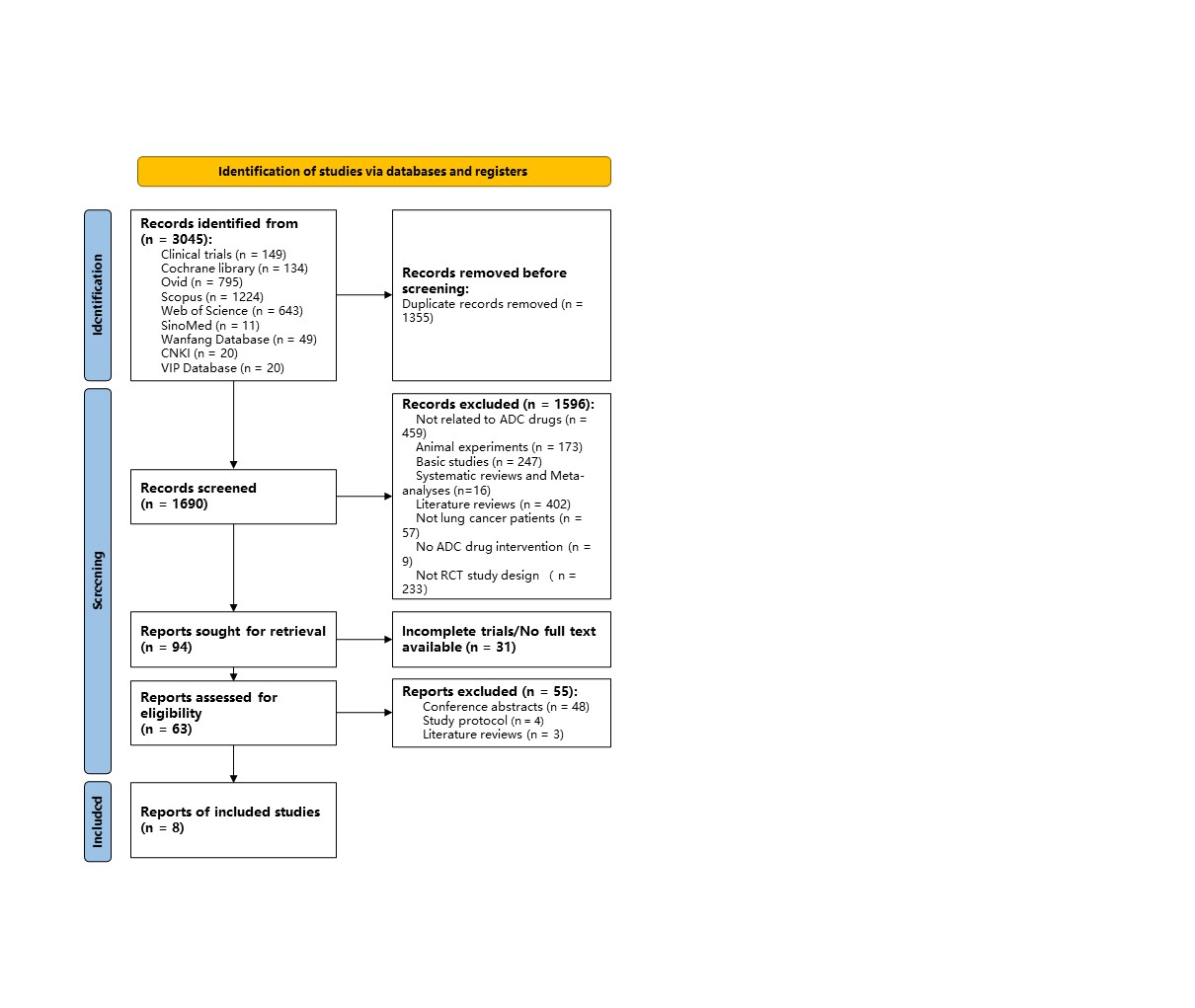


Fig S1. RCT of ADC drugs in Lung Cancer Patients Literature Inclusion and Exclusion Flow Diagram by human reviewers

Table S1. Inclusion-Exclusion Accuracy in ADC drug Title-Abstract Screening

| **Model** | **Accuracy** | **Recall** | **Precision** |
| --- | --- | --- | --- |
| GPT (GPT-4o) | 99.82% | 97.87% | 98.92% |
| Kimi (moonshot-v1-128k) | 99.53% | 98.94% | 93.00% |
| DeepSeek (deepseek-chat 3.0) | 99.59% | 97.87% | 94.85% |

Table S2. Consistency Rate of Exclusion Reasons in ADC drug Title-Abstract Screening

| **Model** | **Consistency Rate** |
| --- | --- |
| GPT (GPT-4o) | 94.20% |
| Kimi (moonshot-v1-128k) | 91.07% |
| DeepSeek (deepseek-chat 3.0) | 93.31% |

Table S3. Inclusion-Exclusion Accuracy in ADC drug Full-Text Screening

| **Model** | **Accuracy** | **Recall** | **Precision** |
| --- | --- | --- | --- |
| GPT (GPT-4o) | 98.41% | 100.00% | 88.89% |
| Kimi (moonshot-v1-128k) | 90.48% | 100.00% | 57.14% |
| DeepSeek (deepseek-chat 3.0) | 98.41% | 100.00% | 88.89% |
