# Supplementary figures and images for: LitAutoScreener: Development and Validation of an Automated Literature Screening Tool in Evidence-Based Medicine Driven by Large Language Models
Source: Health Data Sci. 2025 Sep 2;5:0322. doi: 10.34133/hds.0322 (PMC12404845; doi:10.34133/hds.0322)

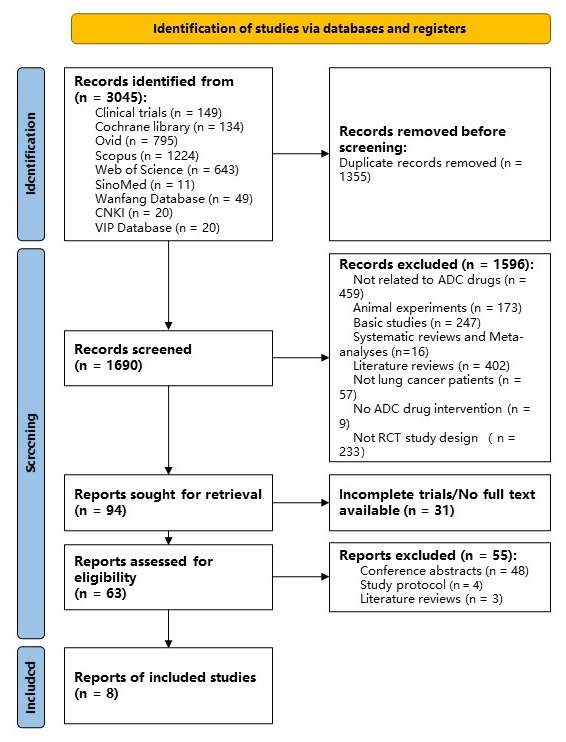

Supplement: Supplementary 1 — Fig. S1 Tables S1 to S3 [file hds.0322.f1.zip › Fig S1.jpg]
